# Magnesium sulfate for 6 vs 24 hours post delivery in patients who received magnesium sulfate for less than 8 hours before birth: a randomized clinical trial

**DOI:** 10.1186/s12884-017-1424-3

**Published:** 2017-07-24

**Authors:** Paulino Vigil-De Gracia, Ricardo Ramirez, Yarelys Durán, Arelis Quintero

**Affiliations:** 1 Complejo Hospitalario de la Caja de Seguro Social, Sistema Nacional de Investigadores, SENACYT, Panamá, Panamá Panamá; 2 Complejo Hospitalario Manuel Amador Guerrero, calle 11, Panamá, Panamá Panamá; 3 Hospital Jose Domingo De Obaldia, San Pablo Viejo 507, Panamá, Panamá Panamá

**Keywords:** Severe preeclampsia, Eclampsia, Magnesium sulfate, Postpartum, Pregnancy, Maternal complication

## Abstract

**Background:**

To compare the benefits of magnesium sulfate for 24 h (h) postpartum versus 6 h postpartum in patients who received magnesium sulfate (Mg) for less than 8 h before birth.

**Methods:**

A randomized, multicenter, open study was conducted between November 2013 and October 2016 in three teaching maternity hospitals in Panama. Pregnant women diagnosed with severe pre-eclampsia or pre-eclampsia with severe features at more than 20 weeks gestation were invited to participate. They were randomized to the following groups in a 1:1 ratio: A- continue Mg for 24 h after birth (control group); and B- receive Mg for 6 h after birth (experimental group). The primary endpoint and variable was seizure (eclampsia) in the first 72 h postpartum.

**Results:**

During the study period, 284 patients agreed to participate in the study; 143 were randomized to receive Mg for 24 h postpartum and 141 to receive Mg for 6 h postpartum. There were no significant differences in the baseline characteristics of the two groups studied. There was no eclampsia in the entire population; therefore, there was no significant difference in the primary variable. Two secondary variables showed a significant difference: time to onset of ambulation, which was 14 h shorter (*p* = 0.0001) in the group that received 6 h of postpartum Mg, and time to initiation of breastfeeding, which was 11 h earlier (*p* = 0.0001) in the group that received 6 h of postpartum Mg. There were not significant differences between the groups with respect to total complications or any particular complication. There were no cases of maternal death.

**Conclusion:**

Maintaining Mg for 6 h postpartum is equally effective in preventing eclampsia as receiving Mg for 24 h postpartum in patients with severe pre-eclampsia who receive less than 8 h of Mg treatment before birth. The onset of maternal ambulation and initiation of breastfeeding are faster in patients who only receive Mg for 6 h postpartum.

**Trial registration:**

The study was registered at clinical-trials.gov, number NCT02317146. Date of registration: December 11, 2014. This study was registered at clinical trials after the beginning of recruitment of patients.

## Background

Hypertension during pregnancy continues to be a pathology with higher maternal morbidity and mortality. These complications may appear during pregnancy, birth or postpartum [1]. Seizures are one of the findings observed in patients with pre-eclampsia and may present in the postpartum period in approximately 2-3% of patients when magnesium sulfate (Mg) is not used [2, 3]. It is well known that the treatment for pre-eclampsia/eclampsia is the interruption of pregnancy and even more categorically so when faced with severe pre-eclampsia or eclampsia [1, 4]. If the cure for pre-eclampsia/eclampsia is the birth of the fetus and elimination of the placenta, then complications should diminish or disappear after birth; but the truth is that postpartum eclampsia occurs in varied percentages ranging from 11 to 44% of all cases of eclampsia [5]. To avoid eclampsia during the postpartum period, Mg is used. However, there are doubts about the utility of using Mg in the postpartum period in women with severe preeclampsia [6]. One arm of The Magpie trial (MAGnesium sulphate for Prevention of Eclampsia) did not show superiority for Mg when first used postpartum [7]. Recently, the data from a randomized study showed no benefit to continuing Mg postpartum when patients with severe pre-eclampsia received more than 8 h (h) of Mg before birth [8]. However, many patients receive less than 8 h of Mg before birth, and the remedy is to use Mg for 24 h postpartum [1, 3–7]. This practice does not have sufficient evidence and is therefore questioned [6]. It is important to consider the secondary effects related to post-partum magnesium use as delay in patient ambulation and breast feeding; other known complications include maternal somnolence, muscular weakness and magnesium toxicity.

The main objective of this research is to compare the benefits of Mg for 24 h postpartum versus 6 h postpartum in patients who received the drug for less than 8 h before birth.

## Methods

### Participants and study design

A randomized, multicenter, open study was conducted between November 2013 and October 2016 in three teaching maternity hospitals in Panama. These maternity units had experience in the management of severe pre-eclampsia. Verbal and written consent to participate was necessary in all women. The ethical committee of “Complejo Hospitalario Dr. Arnulfo Arias Madrid” approved this research. The other hospitals accepted the research.

Pregnant women diagnosed with severe pre-eclampsia or pre-eclampsia with severe features at more than 20 weeks gestation were invited to participate in the MAG-PP (**MAG**nesium-**P**ost **P**artum) study.

Severe pre-eclampsia was diagnosed when a patient had arterial pressures of 140/90 mmHg or higher at least twice separated by 4 h with or without proteinuria ≥0.3 g and had one or more of the following clinical criteria: severe hypertension (160/110 mmHg), neurological symptoms, epigastric pain, or laboratory abnormalities (increase in creatinine, thrombocytopenia, elevation of liver enzymes, changes in clotting times other than HELLP syndrome) [4, 5] When the woman was known to have chronic hypertension and had one of the aforementioned criteria plus the presence of proteinuria ≥0.3 g, it was considered pre-eclampsia with severe features [4, 5].

Exclusion criteria were eclampsia before delivery, HELLP syndrome, epilepsy, and pre-eclampsia with additional pathology such as renal failure, acute pulmonary edema, decompensated diabetes mellitus, autoimmune diseases, or hypertensive encephalopathy.

Eclampsia was defined as the presence of a generalized clonic/tonic seizure when no other cause could be confirmed.

Postpartum hemorrhage was defined as postpartum bleeding greater than 500 ml or post caesarean section bleeding greater than 1000 ml.

Respiratory insufficiency was defined as onset of shortness of breath as demonstrated by tachypnea or dyspnea or an oxygen saturation less than 90%.

The ethics committee and teaching and research authorities of the three hospitals approved the study before commencing recruitment.

### Randomization and masking

After obtaining oral and signed consent to participate in the MAG-PP study, patients were assigned to one of two study groups in a 1:1 ratio: A- continue Mg for 24 h after birth (control group), or B- receive Mg after birth for 6 h (experimental group). Randomization was done with a computerized program using blocks of 4, and sealed envelopes were used for the results of the randomization. The coordinator or investigator of each hospital did not have access to the randomization sequence.

All patients were advised of the benefits and risks of continuing postpartum Mg or stopping after 6 h.

### Interventions

Any patient with severe pre-eclampsia or pre-eclampsia with severe features who had received less than 8 h of Mg for seizure prophylaxis before birth was a candidate to enter the study. The research was explained to candidate patients who agreed to be randomized at birth or immediately postpartum.

All patients received the same management and monitoring (the hospital had similar routine), except that group B was only maintained on Mg for 6 h postpartum. The patients who received Mg for 24 h postpartum maintained a bladder catheter and bed rest. The patients who received Mg for 6 h were allowed to ambulate and breastfeed as tolerated after 6 h. The magnesium sulfate was administrated Intravenously by means of an infusion pump in each hospital. When systolic blood pressures of 160 mmHg or more or diastolic blood pressures of 110 mmHg or more occurred, the following was administered according to hospital availability or the preferences of the treating physician: hydralazine 5 mg every 20 min, up to a maximum of 5 doses if needed; labetalol 20 mg, and the dose was doubled if hypertension persisted 20 min later, and if a third, fourth or fifth dose was required, then 80 mg was given; or the third option was nifedipine 10 mg orally every 20 min for up to 5 doses until the blood pressure was lowered. The use of drugs for elevated postpartum blood pressures in patients without hypertensive crisis was permitted at the discretion of the physicians responsible for the patients. Maternal complications, if any, were recorded.

In our hospitals breastfeeding is allowed in patients with cesarean from 6 h postpartum, in patients with magnesium sulfate is allowed after 24 h when magnesium has been removed.

### Statistical analysis

The primary endpoint and variable was seizure (eclampsia) in the first 72 h postpartum. The secondary variables evaluated were complications such as postpartum bleeding, respiratory distress, maternal death, total time on Mg prior to birth, postpartum convulsion time, number of seizures, admission to the intensive care unit due to a complication, use of postpartum antihypertensives, use of antihypertensives for hypertensive crises, time to onset of ambulation, and breastfeeding start time.

There are no previous similar studies, and it is difficult to use an odds ratio (OR) to calculate an adequate sample, so we decided to use the MAGPIE study [7] in which countries from Latin America participated, which showed that the incidence of eclampsia in the group treated with Mg was 0.8%. Assuming a sample in which the incidence of eclampsia was similar for each group, with a maximum of one patient with eclampsia per group, we could make the comparison. If the experimental group (6 h postpartum Mg) had two patients with eclampsia prior to accruing the desired sample size, the study was to be stopped. Assuming an incidence of seizure of 0.8%, one patient with eclampsia would be present in a sample of 125 patients with severe pre-eclampsia per group. Adding 15% to account for possible protocol alterations or losses, the number of patients should be 286 (143 per group).

The baseline characteristics of the participants in each group were compared with Student’s t test for continuous variables with a normal distribution. Dichotomous variables were compared using Fisher’s exact test and the chi-squared test when appropriate. Values ​​of p less than 0.05 were considered significant.

Statistical analysis was performed with Epi Info software version 7 (Centers for Disease Control and Prevention, Atlanta, GA) under the intent-to-treat principle.

## Results

**Fig. 1:**
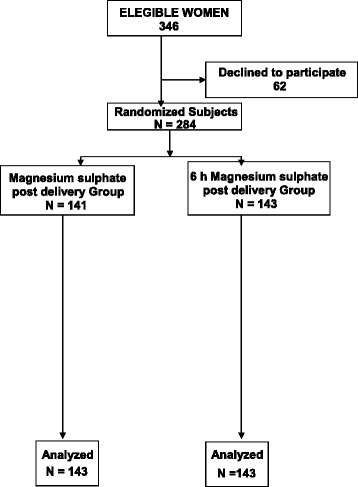
Eligible women, randomization and follow-up of the clinical trial

During the study period, 284 patients in three maternity hospitals in Panama agreed to participate in the MAG-PP study. A total of 143 patients were randomized to receive 24 h of postpartum Mg, and 141 were randomized to receive 6 h of postpartum Mg. Figure 1 shows the study profile and its randomization.

Table 1 shows the baseline characteristics of the study population. There were no significant differences. All patients received 4 g of Mg infusion, plus they received an average of 4 h of Mg before birth. Another interesting baseline finding is that 66% of the population ultimately underwent a cesarean section. The time of postpartum hospitalization was 3 days. One hundred forty-seven women were primigravida without difference by group. Reasons for cesarean section were: fetal compromise, anterior cesarean section, unfavorable cervix, lack of progress in labor without difference by group.

**Table 1:** Patient characteristics at randomization

	24 h Magnesium Sulphate	6 h Magnesium Sulphate	*p* value
	Post Delivery Group	Post Delivery Group	
	*N* = 143	*N* = 141	
Hypertensive disorder N (%)			
Severe Preeclampsia	115 (80.5)	116 (82.3)	0.68
Superimposed preeclampsia^a^	28 (19.5)	25 (17.7)	
Age - SD (year)	28.3 ± 7.5	28.7 ± 6.8	0.65
Parity	2.2 (1.6)	2.5 (1.7)	0.23
Mean Gestational Age (weeks)	36.2 (2.8)	36.1 (3.1)	0.73
Symptoms^b^ n (%)	88 (61.5)	86 (61)	0.92
SBP mmHg, Mean (SD)	155.2 ± 13.1	158.0 ± 17.1	0.24
DBP mmHg, Mean (SD)	100.0 ± 8.9	102.3 ± 10.8	0.14
Hours with magnesium sulfate after loading dose to birth h (SD)	4.6 (2.3)	3.9 (3.8)	0.32
Cesarean Section, N (%)	92 (64.3)	96 (68.0)	0.50

^a^Chronic blood pressure with severe features of superimposed preeclampsia

^b^One or more symptoms: Severe headache, visual disturbances, epigastric pain

There was no eclampsia (seizure) in the entire population. Therefore, there was no significant difference in the primary variable (Table 2). There were also no significant differences between the groups with respect to total complications or any particular complication. Three secondary variables showed significant differences: 1- the diuresis measured between 6 and 12 h postpartum was greater in the group that continued Mg for 24 h; 2- the time to onset of ambulation was 14 h shorter (*p* = 0.001) in the group that received Mg for 6 h postpartum; and 3- the time to initiation of breastfeeding was 11 h earlier (*p* = 0.001) in the group that received Mg for 6 h postpartum.

**Table 2:** Main results postpartum according to treatment group

	24 h Magnesium Sulphate	6 h Magnesium Sulphate	*p* value
	Post Delivery Group	Post Delivery Group	
	*N* = 143	*N* = 141	
Convulsion (eclampsia), N (%)	0	0	1.00
Total complications, N (%)	32 (22.3)	30 (21.2)	0.76
Haemorrhage, N (%)	2 (1.3)	1 (0.7)	0.67
Respiratory depression, N (%)	1 (0.7)	1 (0.7)	0.50
Severe hypertension, N (%)^a^	30 (21.0)	29 (20.2)	0.60
Orin post partum (ml) from 6 to 12 hours ml (SD)	196.6 (87)	150.0 (107.8)	0.003
Time to start ambulation, h (SD)	24.9 ± 3.9	10.9. ± 5.3	0.001
Time to start breastfeeding, h (SD)	36.5 ± 16.8	25.7± 19.8	0.001

^a^Severe Hypertension: Diastolic blood pressure ≥ 110 mmHg and / or Systolic blood pressure ≥ 160 mmHg

There were no cases of maternal death, and all patients were discharged to their homes in good general condition.

## Discussion

The main objective of this research is to compare the benefits of Mg for 24 h postpartum versus 6 h postpartum in patients who received the drug for less than 8 h before birth. The findings from this randomized clinical study show that maintaining Mg for 24 h postpartum yields similar result to receiving Mg for only 6 h postpartum in patients with severe pre-eclampsia who received treatment with Mg infusion for less than 8 h before birth. Therefore, maintaining Mg for 24 h postpartum in patients with severe preeclampsia who received less than 8 h of Mg prior to birth is no better than just maintaining Mg for 6 h postpartum.

According to the results of randomized clinical studies, receiving Mg leads to an eclampsia frequency of 0.6%, with a range from 0.3 to 0.9% [3]. That is, in patients with severe pre-eclampsia in hospitals, receiving Mg represents a seizure risk ranging from 0.3 to 0.9%. All the studies reviewed administered Mg beginning at diagnosis in the antepartum period and maintained treatment for 24 h postpartum; most studies start Mg during labor. Our study shows that after starting infusion and receiving treatment with Mg (4 g on average) for less than 8 h prior to birth, continuing the Mg for 24 h postpartum is no better than just maintaining it for 6 h postpartum. Interestingly, the current study does not report any cases of eclampsia, contrary to expectations. In patients treated with Mg ante- and postpartum, we expected at least two cases of eclampsia among 280 cases of severe pre-eclampsia, according to the results of the MAGPIE study [7]. If the treatment were not adequate in the experimental group, two to three cases of eclampsia were expected [3, 7]. The interesting result was that there were no cases of eclampsia in either the control group or experimental group. That is, the effectiveness of either strategy was 100%. However, neither of these two regimens has been strictly investigated in previous studies because only a particular group has been investigated, i.e., those patients who received low doses of Mg for very short courses before undergoing cesarean section or childbirth.

Approximately 80% of cases of eclampsia occur during pregnancy or intrapartum, and only an average of 20% of cases occur postpartum [5, 9]. This timing means that our main strategy for avoiding eclampsia should focus on the pregnant woman and the moment of birth. Therefore, delivery of the fetus is the main strategy to treat eclampsia [10]. The two known strategies for preventing eclampsia are pregnancy discontinuation and the use of Mg [4, 5, 7]. Thus it seems that if we have used the minimum effective dose of Mg and we interrupt the pregnancy, then the administration of Mg postpartum is not justified. In addition, any postpartum strategy would be focused on preventing only approximately 20% of cases of eclampsia. We do not know the effective minimum dose of Mg; however, our study shows that a 4 g infusion plus an average of 4 g before birth and 6 g postpartum (14 g total) is sufficient to avoid eclampsia; 32 g of Mg was used in the control group.

On the other hand, the secondary objectives such as postpartum hemorrhage, respiratory depression, need for antihypertensive drugs to control blood pressure, severe hypertension, hypertensive encephalopathy, and syncope, among others, showed no significant differences between giving Mg for 6 or 24 h postpartum. Possibly, the similarity between these secondary results occurs because Mg was used in both groups (14 g in the experimental group and 32 g in the control group). For all of the above outcomes, when a patient receives Mg for less than 8 h antepartum, maintaining it for 6 h is already sufficient to avoid eclampsia; however, the side effects are not increased by maintaining it for 24 h.

In many hospitals around the world, as in Panama, patients receiving postpartum Mg are kept in bed for 24 h. In addition, this restriction makes difficult or prevents breastfeeding during this time. As expected, in this study, the group that received postpartum Mg for only 6 h started to ambulate significantly earlier, and they also started breastfeeding sooner. These two findings suggest a great benefit to the mother and her child. In addition, the bladder catheter is maintained for only 6 h. It is also more expensive to maintain Mg longer and requires additional personnel for surveillance purposes [11–13].

### Strengths

This study is a randomized multicenter study in a country with a high frequency of pre-eclampsia/eclampsia. In addition, the methodology is simple and easy to replicate.

### Limitations

The main weakness of this study is that it was not double-blind. However, there was no eclampsia in the whole study population, probably because of the effectiveness of both treatments.

In this study, there was no eclampsia, and there were no maternal deaths. Therefore, it appears that there are more benefits for the mother and her child to using postpartum Mg for only 6 h instead of 24 h in severe pre-eclampsia, provided that patients have received less than 8 h of Mg prior to birth.

## Conclusions

Patients diagnosed with severe pre-eclampsia and treated with Mg for less than 8 h before birth can benefit from receiving Mg for only for 6 h postpartum because this treatment yields the same protection against eclampsia and also results in earlier onset of ambulation and breastfeeding. Studies with greater sample are necessary to corroborate these findings.

## Abbreviations

HELLP: Hemolysis, elevated liver enzymes, and low platelet count
MAGPIE: Magnesium sulphate for prevention of eclampsia
MAG-PP: Magnesium postpartum
Mg: Magnesium sulfate
